# 2-[4-(2-Chloro­acet­yl)phen­yl]-2-methyl-1-(pyrrolidin-1-yl)propan-1-one

**DOI:** 10.1107/S1600536813020175

**Published:** 2013-07-27

**Authors:** Dong-mei Ren

**Affiliations:** a Security and Environment Engineering College, Capital University of Economics and Business, Beijing 10070, People’s Republic of China

## Abstract

The asymmetric unit of the title compound, C_16_H_20_ClNO_2_, contains two mol­ecules in which the dihedral angles between the benzene ring and the plane of the amide unit are 77.4 (1) and 81.1 (1)°. In both mol­ecules, the five-membered ring adopts an envelope conformation with one of the β-C atoms as the flap. In the crystal, mol­ecules are connected *via* C—H⋯O hydrogen bonds, forming chains along the *b*-axis direction. These chains are further linked by C—H⋯π inter­actions, forming a three-dimensional network.

## Related literature

For background to applications of the title compound, see: Krauss *et al.* (2001 ▶). For the synthetic procedure of the title compound, see: Krauss *et al.* (1995 ▶).
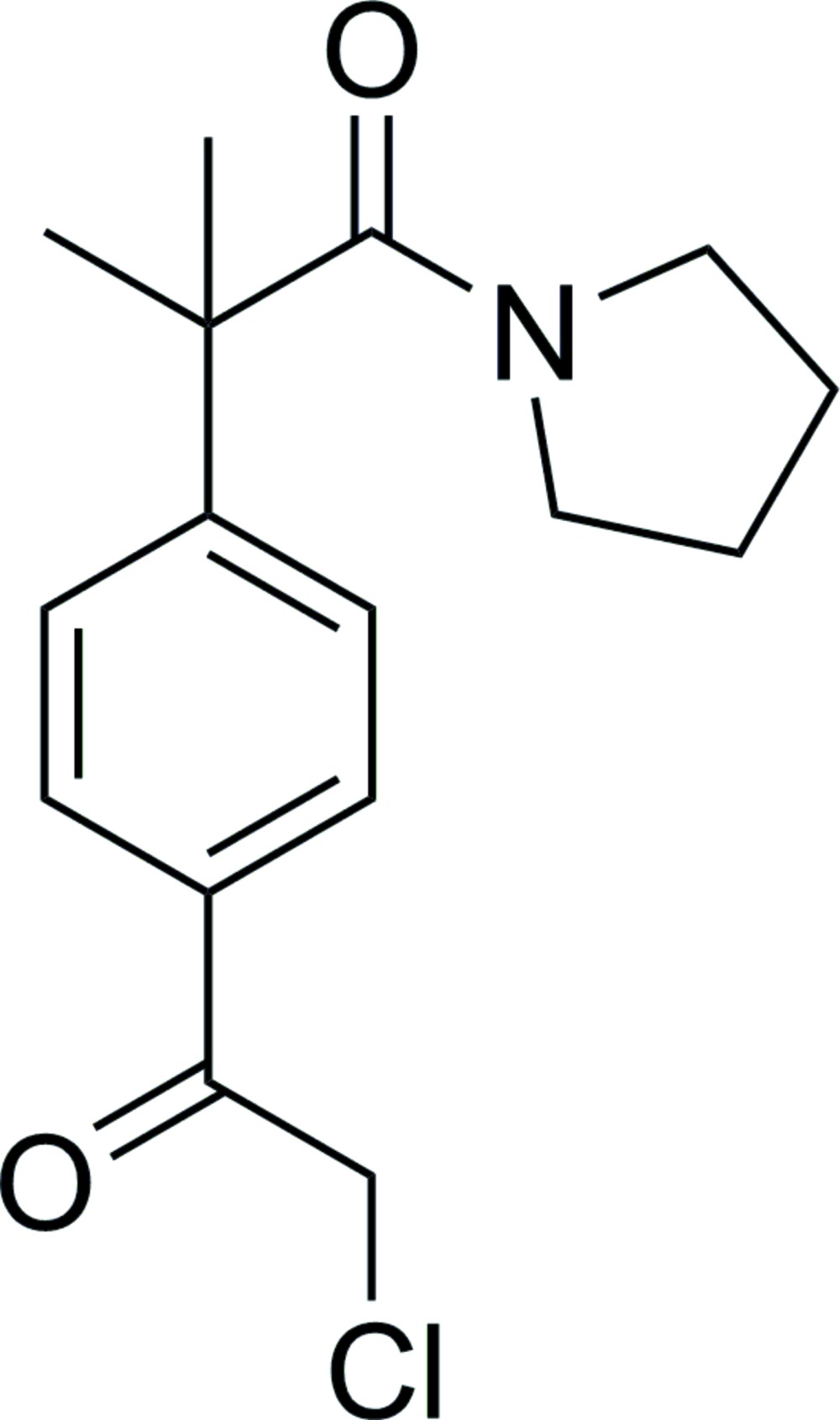


## Experimental

### 

#### Crystal data

C_16_H_20_ClNO_2_
*M* *_r_* = 293.78
Monoclinic, 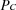

*a* = 8.7380 (17) Å
*b* = 6.1660 (12) Å
*c* = 28.670 (6) Å
β = 95.95 (3)°
*V* = 1536.4 (5) Å^3^
*Z* = 4
Mo *K*α radiation
μ = 0.25 mm^−1^
*T* = 293 K
0.30 × 0.20 × 0.10 mm

#### Data collection

Enraf–Nonius CAD-4 diffractometer
Absorption correction: ψ scan (North *et al.*, 1968 ▶) *T*_min_ = 0.929, *T*_max_ = 0.976
5766 measured reflections
3048 independent reflections
1538 reflections with *I* > 2σ(*I*)
*R*_int_ = 0.093
3 standard reflections every 200 reflections intensity decay: 1%

#### Refinement

*R*[*F* ^2^ > 2σ(*F* ^2^)] = 0.060
*wR*(*F* ^2^) = 0.069
*S* = 0.92
3048 reflections
361 parameters
2 restraints
H-atom parameters constrained
Δρ_max_ = 0.16 e Å^−3^
Δρ_min_ = −0.19 e Å^−3^
Absolute structure: Flack (1983 ▶), 200 Friedel pairs
Absolute structure parameter: 0.06 (7)

### 

Data collection: *CAD-4 Software* (Enraf–Nonius, 1985 ▶); cell refinement: *CAD-4 Software*; data reduction: *XCAD4* (Harms & Wocadlo, 1995 ▶); program(s) used to solve structure: *SHELXS97* (Sheldrick, 2008 ▶); program(s) used to refine structure: *SHELXS97* (Sheldrick, 2008 ▶); molecular graphics: *SHELXTL* (Sheldrick, 2008 ▶); software used to prepare material for publication: *SHELXTL*.

## Supplementary Material

Crystal structure: contains datablock(s) I, RDM. DOI: 10.1107/S1600536813020175/vm2196sup1.cif

Structure factors: contains datablock(s) I. DOI: 10.1107/S1600536813020175/vm2196Isup2.hkl

Supplementary material file. DOI: 10.1107/S1600536813020175/vm2196Isup3.cml

Additional supplementary materials:  crystallographic information; 3D view; checkCIF report

## Figures and Tables

**Table 1:** Hydrogen-bond geometry (Å, °) *Cg*2 and *Cg*4 are the centroids of the C9–C14 and C25–C30 rings, respectively.

*D*—H⋯*A*	*D*—H	H⋯*A*	*D*⋯*A*	*D*—H⋯*A*
C4—H4*A*⋯O1^i^	0.97	2.53	3.427 (9)	153
C32—H32*B*⋯O1^i^	0.97	2.35	3.136 (8)	138
C18—H18*B*⋯*Cg*4^ii^	0.97	2.73	3.448 (7)	131
C4—H4*B*⋯*Cg*2	0.97	2.89	3.728 (9)	145
C23—H23*B*⋯*Cg*2^iii^	0.96	2.98	3.932 (6)	171

Symmetry codes: (i) 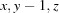; (ii) 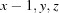; (iii) 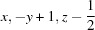.

**Fig. 1.**
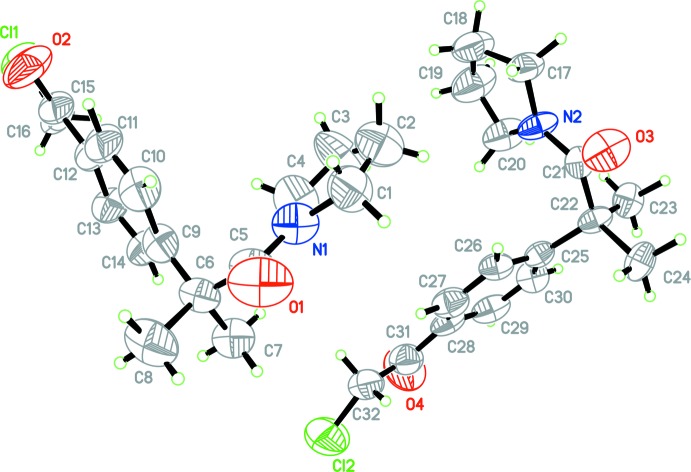
The molecular structure of (I), with the atom-numbering scheme. Displacement ellipsoids are drawn at the 50% probability level.

**Fig. 2.**
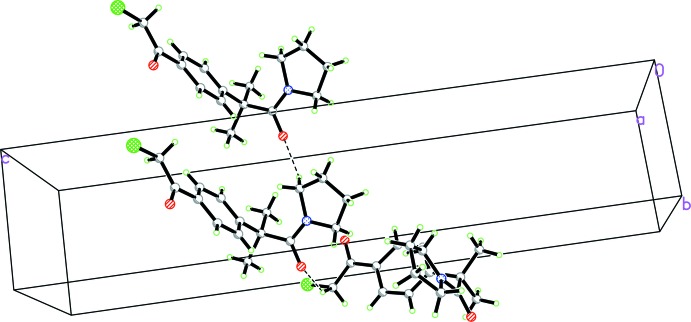
A packing diagram of (I) showing C—H···O interactions.

## supplementary crystallographic information

### Comment

The title compound (I) is an important intermediate in the synthesis of [(piperidinoalkanoyl)phenyl]propionates, which can be utilized to synthesize antihistaminics (Krauss *et al.*, 2001).

The molecular structure of (I) is shown in Fig. 1. There is a intermolecular contact C—H···O in the title compound, forming molecular chains (Table 1, Fig. 2). The crystal packing is further controlled by C—H···π interactions [C18—H18B···*Cg*4 distance of 2.730 Å, C4—H4B···*Cg*2 distance of 2.890 Å and C23—H23B···*Cg2* distance of 2.980 Å (*Cg*4 and *Cg*2 are the centroids of the rings defined by the atoms C25—C30 and C9—C14, respectively)].

The dihedral angles between the benzene ring and the plane of the amide are 77.4 (1)° and 81.1 (1)°, respectively. The conformation of 5-memebred rings are both envelope with the tip atoms C3 and C18, respectively.

### Experimental

The title compound, (I) was prepared by a method reported in literature (Krauss *et al.*, 1995). The crystals were obtained by dissolving (I) (0.1 g) in methanol (30 ml) and evaporating the solvent slowly at room temperature for about 8 d.

### Refinement

All H atoms were positioned geometrically and constrained to ride on their parent atoms, with C—H = 0.93 Å for aromatic H, 0.96 Å for alkyl H and 0.97 Å for other H, respectively. The *U*_iso_(H) = *xU*_eq_(C), where *x* = 1.2 for aromatic H and *x* = 1.5 for other H.

### Crystal data

**Table:**

C_16_H_20_ClNO_2_	*F*(000) = 624
*M**_r_* = 293.78	*D*_x_ = 1.270 Mg m^−^^3^
Monoclinic, *P**c*	Mo *K*α radiation, λ = 0.71073 Å
Hall symbol: P -2yc	Cell parameters from 25 reflections
*a* = 8.7380 (17) Å	θ = 9–12°
*b* = 6.1660 (12) Å	µ = 0.25 mm^−^^1^
*c* = 28.670 (6) Å	*T* = 293 K
β = 95.95 (3)°	Block, colourless
*V* = 1536.4 (5) Å^3^	0.30 × 0.20 × 0.10 mm
*Z* = 4	

### Data collection

**Table:**

Enraf–Nonius CAD-4 diffractometer	1538 reflections with *I* > 2σ(*I*)
Radiation source: fine-focus sealed tube	*R*_int_ = 0.093
Graphite monochromator	θ_max_ = 25.5°, θ_min_ = 1.4°
ω/2θ scans	*h* = 0→10
Absorption correction: ψ scan (North *et al.*, 1968)	*k* = −7→7
*T*_min_ = 0.929, *T*_max_ = 0.976	*l* = −34→34
5766 measured reflections	3 standard reflections every 200 reflections
3048 independent reflections	intensity decay: 1%

### Refinement

**Table:**

Refinement on *F*^2^	Secondary atom site location: difference Fourier map
Least-squares matrix: full	Hydrogen site location: inferred from neighbouring sites
*R*[*F*^2^ > 2σ(*F*^2^)] = 0.060	H-atom parameters constrained
*wR*(*F*^2^) = 0.069	*w* = 1/[σ^2^(*F*_o_^2^) + (0.*P*)^2^] where *P* = (*F*_o_^2^ + 2*F*_c_^2^)/3
*S* = 0.92	(Δ/σ)_max_ < 0.001
3048 reflections	Δρ_max_ = 0.16 e Å^−^^3^
361 parameters	Δρ_min_ = −0.19 e Å^−^^3^
2 restraints	Absolute structure: Flack (1983), 200 Friedel pairs
Primary atom site location: structure-invariant direct methods	Flack parameter: 0.06 (7)

### Special details

**Table:**

Geometry. All e.s.d.'s (except the e.s.d. in the dihedral angle between two l.s. planes) are estimated using the full covariance matrix. The cell e.s.d.'s are taken into account individually in the estimation of e.s.d.'s in distances, angles and torsion angles; correlations between e.s.d.'s in cell parameters are only used when they are defined by crystal symmetry. An approximate (isotropic) treatment of cell e.s.d.'s is used for estimating e.s.d.'s involving l.s. planes.
Refinement. Refinement of *F*^2^ against ALL reflections. The weighted *R*-factor *wR* and goodness of fit *S* are based on *F*^2^, conventional *R*-factors *R* are based on *F*, with *F* set to zero for negative *F*^2^. The threshold expression of *F*^2^ > σ(*F*^2^) is used only for calculating *R*-factors(gt) *etc*. and is not relevant to the choice of reflections for refinement. *R*-factors based on *F*^2^ are statistically about twice as large as those based on *F*, and *R*- factors based on ALL data will be even larger.

### Fractional atomic coordinates and isotropic or equivalent isotropic displacement parameters (Å^2^)

**Table:**

	*x*	*y*	*z*	*U*_iso_*/*U*_eq_	
Cl1	−0.2012 (2)	0.1803 (4)	0.78932 (8)	0.1097 (8)	
O1	0.4889 (5)	0.9996 (8)	0.5934 (2)	0.0921 (17)	
N1	0.3621 (6)	0.7027 (9)	0.5710 (2)	0.0691 (16)	
C1	0.3014 (8)	0.8012 (16)	0.5260 (2)	0.088 (2)	
H1A	0.3851	0.8359	0.5076	0.105*	
H1B	0.2463	0.9339	0.5315	0.105*	
O2	−0.1115 (6)	0.5903 (12)	0.75207 (18)	0.116 (2)	
C2	0.1938 (8)	0.6382 (13)	0.5003 (2)	0.092 (2)	
H2A	0.0912	0.6979	0.4935	0.110*	
H2B	0.2316	0.5932	0.4712	0.110*	
C3	0.1942 (9)	0.4580 (16)	0.5331 (3)	0.116 (3)	
H3A	0.0911	0.4409	0.5422	0.140*	
H3B	0.2194	0.3264	0.5170	0.140*	
C4	0.2949 (9)	0.4789 (11)	0.5729 (3)	0.086 (2)	
H4A	0.3753	0.3701	0.5736	0.103*	
H4B	0.2413	0.4623	0.6007	0.103*	
C5	0.4534 (7)	0.8096 (11)	0.6038 (3)	0.0638 (18)	
C6	0.5056 (6)	0.7068 (10)	0.6500 (2)	0.0571 (17)	
C7	0.6227 (6)	0.5225 (11)	0.6410 (2)	0.0712 (19)	
H7A	0.5714	0.4130	0.6214	0.107*	
H7B	0.6624	0.4591	0.6704	0.107*	
H7C	0.7059	0.5824	0.6258	0.107*	
C8	0.5976 (8)	0.8805 (11)	0.6806 (3)	0.090 (3)	
H8A	0.5305	0.9979	0.6868	0.136*	
H8B	0.6801	0.9345	0.6641	0.136*	
H8C	0.6392	0.8164	0.7097	0.136*	
C9	0.3701 (7)	0.6353 (10)	0.6749 (2)	0.0584 (18)	
C10	0.2419 (7)	0.7633 (11)	0.6742 (2)	0.0719 (19)	
H10A	0.2380	0.8938	0.6579	0.086*	
C11	0.1172 (7)	0.7011 (13)	0.6976 (2)	0.074 (2)	
H11A	0.0315	0.7910	0.6967	0.089*	
C12	0.1183 (7)	0.5080 (11)	0.7223 (2)	0.0623 (18)	
C13	0.2467 (6)	0.3841 (11)	0.7243 (2)	0.0655 (18)	
H13A	0.2527	0.2583	0.7423	0.079*	
C14	0.3707 (7)	0.4430 (10)	0.6996 (2)	0.0589 (17)	
H14A	0.4549	0.3507	0.6999	0.071*	
C15	−0.0202 (8)	0.4508 (12)	0.7470 (3)	0.070 (2)	
C16	−0.0287 (7)	0.2273 (13)	0.7645 (2)	0.085 (2)	
H16A	0.0576	0.2008	0.7879	0.102*	
H16B	−0.0212	0.1267	0.7388	0.102*	
Cl2	0.9469 (2)	0.0349 (6)	0.60136 (9)	0.1388 (12)	
O3	0.2684 (5)	0.5797 (7)	0.34112 (17)	0.0727 (14)	
O4	0.8000 (6)	−0.2310 (11)	0.5262 (2)	0.118 (2)	
N2	0.1664 (5)	0.3029 (8)	0.37657 (18)	0.0543 (14)	
C17	0.0265 (6)	0.4269 (12)	0.3801 (2)	0.0656 (19)	
H17A	−0.0310	0.4452	0.3496	0.079*	
H17B	0.0498	0.5686	0.3937	0.079*	
C18	−0.0630 (7)	0.2889 (14)	0.4121 (3)	0.082 (2)	
H18A	−0.0577	0.3526	0.4432	0.098*	
H18B	−0.1702	0.2786	0.3996	0.098*	
C19	0.0076 (7)	0.0773 (13)	0.4142 (3)	0.102 (3)	
H19A	−0.0562	−0.0226	0.3946	0.123*	
H19B	0.0151	0.0239	0.4462	0.123*	
C20	0.1608 (6)	0.0860 (10)	0.3983 (3)	0.075 (2)	
H20A	0.1739	−0.0275	0.3757	0.090*	
H20B	0.2399	0.0713	0.4245	0.090*	
C21	0.2787 (6)	0.3879 (9)	0.3547 (2)	0.0419 (13)	
C22	0.4252 (6)	0.2566 (8)	0.3476 (2)	0.0453 (15)	
C23	0.3801 (6)	0.0863 (10)	0.3114 (2)	0.0582 (16)	
H23A	0.4686	0.0007	0.3062	0.087*	
H23B	0.3406	0.1547	0.2825	0.087*	
H23C	0.3024	−0.0055	0.3222	0.087*	
C24	0.5426 (6)	0.4097 (10)	0.32785 (19)	0.0579 (17)	
H24A	0.6337	0.3296	0.3229	0.087*	
H24B	0.5687	0.5248	0.3498	0.087*	
H24C	0.4984	0.4697	0.2986	0.087*	
C25	0.5000 (5)	0.1729 (9)	0.3944 (2)	0.0421 (14)	
C26	0.5181 (5)	0.3049 (11)	0.4325 (2)	0.0515 (15)	
H26A	0.4720	0.4409	0.4304	0.062*	
C27	0.6012 (6)	0.2468 (10)	0.4738 (2)	0.0613 (18)	
H27A	0.6171	0.3459	0.4982	0.074*	
C28	0.6630 (6)	0.0328 (12)	0.4789 (3)	0.0585 (17)	
C29	0.6428 (6)	−0.0986 (11)	0.4393 (2)	0.0636 (18)	
H29A	0.6864	−0.2363	0.4407	0.076*	
C30	0.5624 (6)	−0.0338 (9)	0.3990 (2)	0.0535 (16)	
H30A	0.5485	−0.1295	0.3738	0.064*	
C31	0.7586 (8)	−0.0398 (15)	0.5211 (3)	0.072 (2)	
C32	0.8049 (7)	0.1175 (18)	0.5571 (2)	0.094 (3)	
H32B	0.7144	0.1602	0.5717	0.112*	
H32A	0.8422	0.2453	0.5421	0.112*	

### Atomic displacement parameters (Å^2^)

**Table:**

	*U*^11^	*U*^22^	*U*^33^	*U*^12^	*U*^13^	*U*^23^
Cl1	0.0799 (12)	0.1040 (16)	0.150 (2)	−0.0129 (14)	0.0365 (12)	−0.0288 (18)
O1	0.089 (3)	0.035 (2)	0.150 (5)	−0.015 (2)	0.002 (3)	0.012 (3)
N1	0.073 (4)	0.041 (3)	0.094 (5)	−0.013 (3)	0.009 (3)	0.014 (3)
C1	0.086 (5)	0.100 (6)	0.078 (6)	−0.023 (5)	0.007 (4)	0.024 (5)
O2	0.108 (4)	0.134 (6)	0.113 (4)	0.076 (4)	0.045 (3)	0.035 (4)
C2	0.097 (6)	0.073 (6)	0.102 (6)	0.004 (5)	−0.005 (5)	0.016 (5)
C3	0.133 (7)	0.097 (7)	0.106 (7)	−0.033 (6)	−0.046 (6)	0.001 (6)
C4	0.124 (6)	0.035 (4)	0.097 (6)	−0.017 (4)	0.000 (5)	0.008 (4)
C5	0.056 (4)	0.040 (4)	0.093 (5)	0.001 (4)	−0.003 (3)	−0.010 (4)
C6	0.055 (4)	0.036 (3)	0.077 (5)	0.002 (3)	−0.006 (3)	−0.006 (4)
C7	0.077 (4)	0.044 (4)	0.093 (5)	0.007 (4)	0.007 (4)	0.000 (4)
C8	0.097 (6)	0.045 (4)	0.123 (7)	−0.004 (4)	−0.017 (5)	−0.022 (5)
C9	0.074 (4)	0.034 (3)	0.065 (4)	0.019 (3)	−0.002 (4)	−0.008 (3)
C10	0.093 (5)	0.038 (4)	0.083 (5)	0.022 (4)	0.004 (4)	−0.002 (4)
C11	0.076 (5)	0.071 (5)	0.075 (6)	0.036 (4)	0.009 (4)	−0.006 (5)
C12	0.066 (4)	0.052 (4)	0.068 (5)	0.025 (4)	0.003 (4)	−0.011 (4)
C13	0.071 (4)	0.051 (4)	0.076 (5)	0.027 (4)	0.010 (4)	0.010 (4)
C14	0.065 (4)	0.036 (4)	0.077 (5)	0.019 (3)	0.015 (3)	−0.002 (3)
C15	0.069 (4)	0.067 (5)	0.074 (5)	0.038 (4)	0.003 (4)	−0.003 (4)
C16	0.072 (4)	0.088 (6)	0.101 (6)	0.009 (5)	0.032 (4)	−0.026 (5)
Cl2	0.0863 (14)	0.218 (3)	0.1052 (16)	0.0096 (19)	−0.0210 (11)	0.042 (2)
O3	0.079 (3)	0.026 (2)	0.115 (4)	0.009 (2)	0.024 (3)	0.009 (3)
O4	0.132 (5)	0.101 (5)	0.114 (5)	0.051 (4)	−0.014 (4)	0.024 (4)
N2	0.037 (2)	0.032 (2)	0.098 (4)	−0.002 (2)	0.021 (3)	0.007 (3)
C17	0.046 (4)	0.070 (5)	0.083 (5)	0.015 (4)	0.016 (3)	0.006 (4)
C18	0.050 (4)	0.092 (6)	0.106 (6)	0.004 (4)	0.020 (4)	0.011 (5)
C19	0.086 (6)	0.083 (6)	0.145 (8)	0.016 (5)	0.046 (6)	0.041 (6)
C20	0.060 (4)	0.038 (4)	0.130 (7)	−0.003 (3)	0.027 (4)	0.019 (4)
C21	0.053 (3)	0.031 (3)	0.042 (3)	−0.001 (3)	0.005 (3)	0.001 (3)
C22	0.035 (3)	0.031 (3)	0.071 (4)	−0.012 (3)	0.013 (3)	−0.003 (3)
C23	0.072 (4)	0.041 (3)	0.063 (4)	0.000 (3)	0.014 (3)	0.005 (4)
C24	0.071 (4)	0.047 (3)	0.060 (4)	−0.008 (3)	0.025 (3)	0.005 (3)
C25	0.038 (3)	0.034 (3)	0.056 (4)	−0.006 (3)	0.013 (3)	−0.002 (3)
C26	0.038 (3)	0.050 (3)	0.067 (4)	0.008 (3)	0.007 (3)	−0.001 (4)
C27	0.050 (3)	0.055 (4)	0.080 (5)	0.000 (3)	0.013 (3)	−0.011 (4)
C28	0.036 (3)	0.058 (4)	0.085 (5)	0.012 (3)	0.023 (3)	0.004 (4)
C29	0.048 (4)	0.037 (3)	0.109 (6)	0.000 (3)	0.023 (4)	0.007 (4)
C30	0.063 (4)	0.030 (3)	0.067 (5)	−0.002 (3)	0.007 (3)	−0.007 (3)
C31	0.059 (4)	0.082 (6)	0.076 (5)	0.004 (4)	0.007 (4)	0.008 (5)
C32	0.055 (4)	0.150 (8)	0.076 (5)	−0.012 (5)	0.006 (4)	0.020 (6)

### Geometric parameters (Å, º)

**Table:**

Cl1—C16	1.756 (6)	Cl2—C32	1.757 (7)
O1—C5	1.256 (8)	O3—C21	1.246 (7)
N1—C5	1.341 (8)	O4—C31	1.237 (9)
N1—C1	1.475 (8)	N2—C21	1.326 (6)
N1—C4	1.502 (8)	N2—C17	1.454 (7)
C1—C2	1.515 (10)	N2—C20	1.479 (8)
C1—H1A	0.9700	C17—C18	1.526 (8)
C1—H1B	0.9700	C17—H17A	0.9700
O2—C15	1.192 (8)	C17—H17B	0.9700
C2—C3	1.455 (11)	C18—C19	1.442 (10)
C2—H2A	0.9700	C18—H18A	0.9700
C2—H2B	0.9700	C18—H18B	0.9700
C3—C4	1.375 (9)	C19—C20	1.459 (7)
C3—H3A	0.9700	C19—H19A	0.9700
C3—H3B	0.9700	C19—H19B	0.9700
C4—H4A	0.9700	C20—H20A	0.9700
C4—H4B	0.9700	C20—H20B	0.9700
C5—C6	1.497 (10)	C21—C22	1.547 (7)
C6—C9	1.510 (8)	C22—C23	1.501 (8)
C6—C8	1.554 (9)	C22—C25	1.521 (8)
C6—C7	1.568 (8)	C22—C24	1.543 (7)
C7—H7A	0.9600	C23—H23A	0.9600
C7—H7B	0.9600	C23—H23B	0.9600
C7—H7C	0.9600	C23—H23C	0.9600
C8—H8A	0.9600	C24—H24A	0.9600
C8—H8B	0.9600	C24—H24B	0.9600
C8—H8C	0.9600	C24—H24C	0.9600
C9—C10	1.369 (7)	C25—C26	1.357 (8)
C9—C14	1.381 (9)	C25—C30	1.387 (7)
C10—C11	1.391 (8)	C26—C27	1.371 (8)
C10—H10A	0.9300	C26—H26A	0.9300
C11—C12	1.384 (9)	C27—C28	1.428 (9)
C11—H11A	0.9300	C27—H27A	0.9300
C12—C13	1.353 (7)	C28—C29	1.390 (9)
C12—C15	1.507 (9)	C28—C31	1.468 (10)
C13—C14	1.401 (8)	C29—C30	1.349 (8)
C13—H13A	0.9300	C29—H29A	0.9300
C14—H14A	0.9300	C30—H30A	0.9300
C15—C16	1.471 (10)	C31—C32	1.442 (11)
C16—H16A	0.9700	C32—H32B	0.9700
C16—H16B	0.9700	C32—H32A	0.9700
			
C5—N1—C1	123.0 (6)	C21—N2—C17	119.6 (5)
C5—N1—C4	129.2 (6)	C21—N2—C20	128.0 (4)
C1—N1—C4	107.7 (6)	C17—N2—C20	112.4 (4)
N1—C1—C2	107.5 (6)	N2—C17—C18	103.6 (5)
N1—C1—H1A	110.2	N2—C17—H17A	111.0
C2—C1—H1A	110.2	C18—C17—H17A	111.0
N1—C1—H1B	110.2	N2—C17—H17B	111.0
C2—C1—H1B	110.2	C18—C17—H17B	111.0
H1A—C1—H1B	108.5	H17A—C17—H17B	109.0
C3—C2—C1	103.5 (6)	C19—C18—C17	106.6 (6)
C3—C2—H2A	111.1	C19—C18—H18A	110.4
C1—C2—H2A	111.1	C17—C18—H18A	110.4
C3—C2—H2B	111.1	C19—C18—H18B	110.4
C1—C2—H2B	111.1	C17—C18—H18B	110.4
H2A—C2—H2B	109.0	H18A—C18—H18B	108.6
C4—C3—C2	115.1 (7)	C18—C19—C20	110.9 (6)
C4—C3—H3A	108.5	C18—C19—H19A	109.5
C2—C3—H3A	108.5	C20—C19—H19A	109.5
C4—C3—H3B	108.5	C18—C19—H19B	109.5
C2—C3—H3B	108.5	C20—C19—H19B	109.5
H3A—C3—H3B	107.5	H19A—C19—H19B	108.1
C3—C4—N1	105.9 (7)	C19—C20—N2	103.7 (5)
C3—C4—H4A	110.5	C19—C20—H20A	111.0
N1—C4—H4A	110.5	N2—C20—H20A	111.0
C3—C4—H4B	110.5	C19—C20—H20B	111.0
N1—C4—H4B	110.5	N2—C20—H20B	111.0
H4A—C4—H4B	108.7	H20A—C20—H20B	109.0
O1—C5—N1	115.9 (7)	O3—C21—N2	119.4 (5)
O1—C5—C6	123.0 (7)	O3—C21—C22	119.3 (5)
N1—C5—C6	121.1 (6)	N2—C21—C22	121.3 (5)
C5—C6—C9	111.1 (5)	C23—C22—C25	115.5 (5)
C5—C6—C8	107.4 (5)	C23—C22—C24	108.1 (5)
C9—C6—C8	108.7 (6)	C25—C22—C24	106.8 (4)
C5—C6—C7	107.8 (6)	C23—C22—C21	107.2 (4)
C9—C6—C7	115.0 (5)	C25—C22—C21	110.3 (5)
C8—C6—C7	106.5 (5)	C24—C22—C21	108.6 (4)
C6—C7—H7A	109.5	C22—C23—H23A	109.5
C6—C7—H7B	109.5	C22—C23—H23B	109.5
H7A—C7—H7B	109.5	H23A—C23—H23B	109.5
C6—C7—H7C	109.5	C22—C23—H23C	109.5
H7A—C7—H7C	109.5	H23A—C23—H23C	109.5
H7B—C7—H7C	109.5	H23B—C23—H23C	109.5
C6—C8—H8A	109.5	C22—C24—H24A	109.5
C6—C8—H8B	109.5	C22—C24—H24B	109.5
H8A—C8—H8B	109.5	H24A—C24—H24B	109.5
C6—C8—H8C	109.5	C22—C24—H24C	109.5
H8A—C8—H8C	109.5	H24A—C24—H24C	109.5
H8B—C8—H8C	109.5	H24B—C24—H24C	109.5
C10—C9—C14	117.5 (6)	C26—C25—C30	117.5 (6)
C10—C9—C6	120.4 (6)	C26—C25—C22	120.7 (5)
C14—C9—C6	122.1 (5)	C30—C25—C22	121.6 (6)
C9—C10—C11	121.0 (6)	C25—C26—C27	123.0 (6)
C9—C10—H10A	119.5	C25—C26—H26A	118.5
C11—C10—H10A	119.5	C27—C26—H26A	118.5
C12—C11—C10	121.3 (6)	C26—C27—C28	119.4 (7)
C12—C11—H11A	119.4	C26—C27—H27A	120.3
C10—C11—H11A	119.4	C28—C27—H27A	120.3
C13—C12—C11	118.0 (6)	C29—C28—C27	116.1 (6)
C13—C12—C15	123.4 (6)	C29—C28—C31	120.6 (6)
C11—C12—C15	118.6 (6)	C27—C28—C31	122.9 (7)
C12—C13—C14	120.9 (6)	C30—C29—C28	122.5 (6)
C12—C13—H13A	119.6	C30—C29—H29A	118.7
C14—C13—H13A	119.6	C28—C29—H29A	118.7
C9—C14—C13	121.3 (6)	C29—C30—C25	121.2 (6)
C9—C14—H14A	119.4	C29—C30—H30A	119.4
C13—C14—H14A	119.4	C25—C30—H30A	119.4
O2—C15—C16	125.2 (7)	O4—C31—C32	120.0 (8)
O2—C15—C12	118.0 (7)	O4—C31—C28	121.4 (8)
C16—C15—C12	116.8 (6)	C32—C31—C28	118.6 (7)
C15—C16—Cl1	111.6 (5)	C31—C32—Cl2	116.5 (7)
C15—C16—H16A	109.3	C31—C32—H32B	108.2
Cl1—C16—H16A	109.3	Cl2—C32—H32B	108.2
C15—C16—H16B	109.3	C31—C32—H32A	108.2
Cl1—C16—H16B	109.3	Cl2—C32—H32A	108.2
H16A—C16—H16B	108.0	H32B—C32—H32A	107.3
			
C5—N1—C1—C2	−175.6 (6)	C21—N2—C17—C18	−173.2 (6)
C4—N1—C1—C2	0.9 (9)	C20—N2—C17—C18	8.0 (7)
N1—C1—C2—C3	2.2 (9)	N2—C17—C18—C19	−15.1 (8)
C1—C2—C3—C4	−5.1 (11)	C17—C18—C19—C20	17.4 (10)
C2—C3—C4—N1	5.7 (11)	C18—C19—C20—N2	−12.2 (10)
C5—N1—C4—C3	172.3 (7)	C21—N2—C20—C19	−176.7 (7)
C1—N1—C4—C3	−3.9 (9)	C17—N2—C20—C19	2.0 (8)
C1—N1—C5—O1	−2.0 (10)	C17—N2—C21—O3	6.2 (9)
C4—N1—C5—O1	−177.7 (7)	C20—N2—C21—O3	−175.2 (7)
C1—N1—C5—C6	176.8 (6)	C17—N2—C21—C22	−176.3 (5)
C4—N1—C5—C6	1.1 (11)	C20—N2—C21—C22	2.3 (9)
O1—C5—C6—C9	121.9 (7)	O3—C21—C22—C23	−110.9 (6)
N1—C5—C6—C9	−56.8 (8)	N2—C21—C22—C23	71.6 (7)
O1—C5—C6—C8	3.2 (10)	O3—C21—C22—C25	122.5 (6)
N1—C5—C6—C8	−175.5 (6)	N2—C21—C22—C25	−55.0 (7)
O1—C5—C6—C7	−111.2 (7)	O3—C21—C22—C24	5.7 (8)
N1—C5—C6—C7	70.0 (8)	N2—C21—C22—C24	−171.8 (5)
C5—C6—C9—C10	−40.4 (8)	C23—C22—C25—C26	−167.0 (4)
C8—C6—C9—C10	77.6 (7)	C24—C22—C25—C26	72.6 (6)
C7—C6—C9—C10	−163.2 (6)	C21—C22—C25—C26	−45.2 (6)
C5—C6—C9—C14	140.6 (6)	C23—C22—C25—C30	17.9 (7)
C8—C6—C9—C14	−101.4 (7)	C24—C22—C25—C30	−102.4 (6)
C7—C6—C9—C14	17.8 (9)	C21—C22—C25—C30	139.7 (5)
C14—C9—C10—C11	0.1 (9)	C30—C25—C26—C27	3.5 (8)
C6—C9—C10—C11	−179.0 (6)	C22—C25—C26—C27	−171.7 (5)
C9—C10—C11—C12	−0.2 (10)	C25—C26—C27—C28	−4.8 (9)
C10—C11—C12—C13	2.2 (10)	C26—C27—C28—C29	4.5 (8)
C10—C11—C12—C15	179.8 (7)	C26—C27—C28—C31	177.4 (6)
C11—C12—C13—C14	−4.1 (10)	C27—C28—C29—C30	−3.6 (9)
C15—C12—C13—C14	178.5 (6)	C31—C28—C29—C30	−176.6 (6)
C10—C9—C14—C13	−2.0 (9)	C28—C29—C30—C25	2.6 (9)
C6—C9—C14—C13	177.0 (6)	C26—C25—C30—C29	−2.3 (8)
C12—C13—C14—C9	4.1 (10)	C22—C25—C30—C29	172.9 (5)
C13—C12—C15—O2	163.2 (7)	C29—C28—C31—O4	−13.5 (10)
C11—C12—C15—O2	−14.2 (10)	C27—C28—C31—O4	174.0 (7)
C13—C12—C15—C16	−14.7 (10)	C29—C28—C31—C32	166.1 (6)
C11—C12—C15—C16	167.9 (7)	C27—C28—C31—C32	−6.4 (9)
O2—C15—C16—Cl1	5.8 (11)	O4—C31—C32—Cl2	11.1 (10)
C12—C15—C16—Cl1	−176.5 (5)	C28—C31—C32—Cl2	−168.6 (5)

### Hydrogen-bond geometry (Å, º)

Cg2 and Cg4 are the centroids of the C9–C14 and C25–C30 rings, respectively.

**Table:**

*D*—H···*A*	*D*—H	H···*A*	*D*···*A*	*D*—H···*A*
C4—H4*A*···O1^i^	0.97	2.53	3.427 (9)	153
C32—H32*B*···O1^i^	0.97	2.35	3.136 (8)	138
C18—H18*B*···*Cg*4^ii^	0.97	2.73	3.448 (7)	131
C4—H4*B*···*Cg*2	0.97	2.89	3.728 (9)	145
C23—H23*B*···*Cg*2^iii^	0.96	2.98	3.932 (6)	171

Symmetry codes: (i) *x*, *y*−1, *z*; (ii) *x*−1, *y*, *z*; (iii) *x*, −*y*+1, *z*−1/2.
